# Immune and Epigenetic Pathways Linking Childhood Adversity and Health Across the Lifespan

**DOI:** 10.3389/fpsyg.2021.788351

**Published:** 2021-11-26

**Authors:** Michelle A. Chen, Angie S. LeRoy, Marzieh Majd, Jonathan Y. Chen, Ryan L. Brown, Lisa M. Christian, Christopher P. Fagundes

**Affiliations:** ^1^Department of Psychological Sciences, Rice University, Houston, TX, United States; ^2^McGovern Medical School, University of Texas Health Science Center at Houston, Houston, TX, United States; ^3^Department of Psychiatry & Behavioral Health and the Institute for Behavioral Medicine Research, The Ohio State University Wexner Medical Center, Columbus, OH, United States; ^4^Department of Behavioral Sciences, The University of Texas MD Anderson Cancer Center, Houston, TX, United States; ^5^Department of Psychiatry & Behavioral Sciences, Baylor College of Medicine, Houston, TX, United States

**Keywords:** childhood adversity, early life stress, immune pathways, epigenetic pathways, inflammation

## Abstract

Childhood adversity is associated with a host of mental and physical health problems across the lifespan. Individuals who have experienced childhood adversity (e.g., child abuse and neglect, family conflict, poor parent/child relationships, low socioeconomic status or extreme poverty) are at a greater risk for morbidity and premature mortality than those not exposed to childhood adversity. Several mechanisms likely contribute to the relationship between childhood adversity and health across the lifespan (e.g., health behaviors, cardiovascular reactivity). In this paper, we review a large body of research within the field of psychoneuroimmunology, demonstrating the relationship between early life stress and alterations of the immune system. We first review the literature demonstrating that childhood adversity is associated with immune dysregulation across different indices, including proinflammatory cytokine production (and its impact on telomere length), illness and infection susceptibility, latent herpesvirus reactivation, and immune response to a tumor. We then summarize the growing literature on how childhood adversity may alter epigenetic processes. Finally, we propose future directions related to this work that have basic and applied implications.

## Introduction

There is ample research demonstrating a link between childhood adversity and a host of mental and physical health problems across the lifespan. Childhood adversity refers to experiences of early life stress, including child abuse and neglect, family conflict, poor parent/child relationships, low socioeconomic status or extreme poverty, and other challenges that place undue stress on an individual during their sensitive developmental periods (i.e., during childhood and adolescence when developmental systems are forming). Individuals who have experienced toxic childhood stress (e.g., childhood abuse, neglect, poverty) are at a greater risk for morbidity, and premature mortality than those not exposed to significant adverse childhood experiences (Dong et al., 2004; Anda et al., 2009; Dube et al., 2009; Miller et al., 2011). The health implications of this data are vast. Multiple mechanisms likely contribute to the link between early-life stress and adult health, repeatedly reported in the epidemiological literature (e.g., health behaviors, cardiovascular reactivity). In this paper, we focus on the immune system. We review a large body of research within the field of psychoneuroimmunology, showing that early life stress is associated with immune system alterations that impact disease risk.

Several models contribute to our understanding of childhood adversity and health. For example, biological embedding models of childhood adversity imply that stressors that occur during sensitive periods of development impact the proper development of immune and other physiological systems and, in turn, make individuals more vulnerable to disease in adulthood (Miller et al., 2011). Mechanistically, childhood adversity affects health, in part, by disrupting the body’s ability to regulate itself. Childhood adversity disrupts the development of the body’s regulatory system, which can lead to immune dysregulation, particularly when facing a stressor later in life (Gunnar et al., 2001, 2003; Ellis et al., 2005). Furthermore, early life stress promotes increased stress sensitivity across the lifespan, leading to further adverse health outcomes later in life. For instance, people who have experienced early life stress have significantly more emotional reactivity (measured by negative affect) to daily-life stress than people who have not experienced early life stress (Glaser et al., 2006). Greater emotional reactivity to daily-life stressors is associated with an exaggerated inflammatory response in those who reported childhood trauma than those who did not report childhood trauma (Carpenter et al., 2010). Early adversity and increased psychological vulnerability in adulthood can also been seen in those with a history of childhood abuse or low SES as they perceive ambiguous situations as more threatening compared to those without a history of childhood abuse or low SES (Chen and Matthews, 2003; Miller et al., 2011). Those who experienced early adversities may find certain events more stressful than others and show greater vulnerability to immune dysregulation during stressful events in adulthood, supporting the notion that severe chronic stressors have long-term consequences on one’s physiology (Miller et al., 2011).

### Overview

We first summarize data showing that adverse childhood experiences are associated with immune dysregulation across many indices, including proinflammatory cytokine production (and its impact on telomere length), illness and infection susceptibility, latent herpesvirus reactivation, and immune response to a tumor. We then review the growing literature concerning how early life stress may alter epigenetic processes. Finally, we propose directions for future work that have basic and applied implications.

## Childhood Adversity and Immune Pathways

### Inflammation

Early life stress negatively affects health by elevating levels of inflammation across the lifespan (Miller et al., 2011; Fagundes et al., 2013a; Schwaiger et al., 2016). Under normal conditions, the local inflammatory response helps the body maintain physical health by killing invaders when the immune system identifies a foreign bacteria or virus. However, conditions of chronic stress lead to elevated systemic, chronic inflammation, which is associated with fatigue, disability, and disease including cardiovascular disease, type II diabetes, Alzheimer’s disease, osteoporosis, periodontal disease, rheumatoid arthritis, and some cancers (Ershler and Keller, 2000; Libby, 2007; Carpenter et al., 2010; Fioranelli et al., 2018; Agorastos et al., 2019). Higher levels of inflammatory markers such as proinflammatory cytokines [e.g., interleukin 6 (IL-6) and tumor necrosis factor-alpha (TNF-α)], C-reactive protein (CRP), and fibrinogen, reliably predict increased morbidity and mortality and decreased quality of life in older adults (Ershler and Keller, 2000; Mills et al., 2005; Kiecolt-Glaser et al., 2010; Proctor et al., 2015; Puzianowska-Kuźnicka et al., 2016; Li et al., 2017). Thus, the measurement of inflammatory biomarkers is important in understanding the pathways by which early life stress affects physical health.

Early life stress increases the risk of elevated inflammation through many mechanisms, including psychological and physiological hypersensitivity to stress. Hypersensitivity to physiological stress disrupts neurobiological development and alters the brain’s autonomic stress response, increasing sympathetic activity and decreasing parasympathetic activity (Heim et al., 2000). This response is consistent with increases in inflammation as norepinephrine promotes the production of proinflammatory cytokines while parasympathetic activity is associated with the cholinergic anti-inflammatory pathway (Bierhaus et al., 2003; Tracey, 2009). In line with this notion, those experiencing childhood maltreatment have been found to have lower parasympathetic activity by measurement of heart-rate variability (Dale et al., 2009; Oosterman et al., 2010). Additionally, children raised in low-SES families showed greater levels of sympathetic activity, as measured by increases in the catecholamine epinephrine (Evans and English, 2002). These autonomic processes have linked early life stress to more pronounced stress-induced HPA axis activity in adulthood, increasing cortisol levels to the point of glucocorticoid insensitivity, which in turn dysregulates the production of proinflammatory cytokines (Heim et al., 2000; Miller et al., 2011).

Early life stress affects the body’s response to stress by signaling proinflammatory pathways that promote inflammation (Schwaiger et al., 2016). Early life stress increases the risk of elevated inflammation through several mechanisms. One model regarding early adversity and heightened inflammation states that early life stress triggers monocytes and macrophages to activate an excessive inflammatory response to microbial stimulation (Miller et al., 2011). Early life stress also triggers immune cell insensitivity to the anti-inflammatory effects of cortisol, causing resistance to inhibitory mechanisms of inflammation (Miller et al., 2011). Another mechanism through which early life stress can elevate systemic inflammation is through increased transcription of nuclear factor kappa B cells (NF-κB), an intracellular signaling molecule that regulates proinflammatory-cytokine gene expression (Straub and Härle, 2005). One possible mechanism explaining the increased transcription of NF-κB is through epigenetic processes that favor the production of inflammatory cytokines (Gillespie et al., 2019).

#### Childhood Socioeconomic Status

There is evidence to show that low socioeconomic status in childhood has an impact on inflammation in adulthood. Lawlor et al. (2005) found that older adults raised in low SES homes in childhood had higher CRP levels than those raised in higher SES homes. Another study showed that adults who grew up in lower SES neighborhoods or whose parents had less education had higher serum levels of CRP and fibrinogen than those who grew up in higher SES neighborhoods or whose parents were more educated (Pollitt et al., 2007). Taylor et al. (2006) also found an association between low childhood SES and elevated CRP levels in adulthood and an association between harsh family environments and elevated CRP levels in adulthood; furthermore, they found that these relationships were mediated by psychosocial functioning and BMI. In another study, Chen et al. (2003) studied how low SES affected adolescents with persistent asthma and found that those in lower SES neighborhoods had higher IL-5 and IFN-γ. Furthermore, several other studies also found associations between low SES in childhood and increased inflammatory markers, such as IL-6, NF-κB, and CRP, in adulthood (Danese et al., 2007; Tabassum et al., 2008; Miller et al., 2009; Phillips et al., 2009). Expanding upon this line of research, Chen et al. (2011) found that in adults who experienced low childhood SES, maternal warmth buffered the association between low childhood SES and pro-inflammatory signaling. Specifically, individuals with low childhood SES who had mothers who expressed high warmth toward them exhibited less IL-6 production and reduced NF-κB activity than individuals with low childhood SES who experienced low maternal warmth. These findings imply that the family environment could have an impact on the relationship between childhood SES and inflammation.

#### Harsh Family Environments and Adverse Childhood Experiences

Researchers have examined the relationship between the family environment (e.g., whether the individual felt cared for, was shown physical affection, was verbally or physically abused, lived with a substance abuser, lived in a well-managed household) and inflammation. For instance, Miller and Chen (2010) found that adolescents who grew up in harsher family environments showed greater IL-6 production in response to a microbial challenge; they also found that they had increased glucocorticoid resistance over time, which prevented cortisol from inhibiting inflammatory marker production. In another study, Chiang et al. (2017) found that in adolescents, there was a significant interaction between abdominal adiposity, IL-6 reactivity, and early adversity (i.e., frequency of conflict, violence, harsh discipline, affectionate behaviors, neglect, and chaos/disorganization in their family environment), where early adversity was associated with higher IL-6 reactivity only among adolescents with higher abdominal adiposity. Using a composite scoring system that incorporated elements of stressful life events, quality of relationships with parents, and presence of verbal abuse, Slopen et al. (2010) found early life adversity was associated with CRP, IL-6, fibrinogen, E-selectin, and sICAM-1, the soluble form of intracellular adhesion molecule-1 (ICAM-1) which facilitates leukocyte migration into tissues, among African Americans, while early life adversity was not associated with CRP, IL-6, fibrinogen, E-selectin, and sICAM-1 among White Americans.

Researchers have similarly investigated the role of adverse childhood experiences, including child abuse and neglect, parental separation or divorce, and living in a violent household on inflammation (Felitti et al., 1998). For instance, Rasmussen et al. (2020) found that 18-year-olds who had been exposed to more adverse childhood experiences showed higher levels of suPAR (a biomarker of immune activity and chronic inflammation) compared to those with less exposure to adverse childhood experiences. Recent work has also demonstrated a significant association between adverse childhood experiences and CRP in adolescents (Kliewer and Robins, 2021) and an association between childhood victimization and CRP in 18-year-old females, but not males (Baldwin et al., 2018). In another study, Lacey et al. (2020) studied how specific adverse childhood experiences affected inflammation and found that 9-year-olds who had experienced parental separation had statistically elevated IL-6 compared to those who had experienced other types of adverse childhood experiences. Other research examining specific types of adverse childhood experiences includes work examining the impact of childhood maltreatment, defined as physical abuse, physical neglect, emotional abuse, emotional neglect, and sexual abuse (Bernstein et al., 2003; Mauritz et al., 2013). For instance, Carpenter et al. (2010) found that in adults without psychiatric disorders, those who self-reported a history of childhood maltreatment showed higher levels of IL-6 and higher increases of IL-6 in response to a lab social stress test compared to those without a history of childhood maltreatment. Kiecolt-Glaser et al. (2011) studied the interaction between caregiver status and childhood maltreatment in older adults and found that childhood maltreatment was independently associated with increased IL-6 and TNF-α levels; however, there was an interaction between caregiver status, childhood maltreatment, and inflammation where the association between childhood maltreatment and TNF-α was augmented in caregivers. In Baumeister et al. (2016) conducted a meta-analysis that showed a relationship between childhood maltreatment and inflammation markers such as IL-6, TNF-α, and CRP. Recently, Ehrlich et al. (2021) found an association between maltreatment and inflammation in 8–12-year-old girls, but there was no association present in boys. In another study, Entringer et al. (2020) reported similar findings; among 3–5-year-olds who were studied prospectively immediately after experiencing maltreatment, they found an association between maltreatment and CRP only in girls but not boys. Most recently, Renna et al. (2021) assessed the relationship between childhood abuse and inflammation trajectories over time and found that those who had experienced abuse in childhood exhibited steeper rises in inflammation across time than those who did not experience any type of abuse.

#### Early Life Stress, Inflammation, and Depression

Early life stress can contribute to a proinflammatory phenotype evident in a subset of individuals with depression. For example, Danese et al. (2008) found that adults with a current diagnosis of major depression and a history of childhood maltreatment were 1.48 times more likely to exhibit elevated CRP levels than depressed patients with no history of maltreatment. In another study, MDD patients with a history of childhood adversity, but not MDD patients without childhood adversity, had higher levels of IL-6 compared to healthy controls (De Punder et al., 2018). Furthermore, Pace et al. (2006) showed that individuals with current major depression and increased early life stress exhibited elevated baseline levels of inflammation coupled with a larger inflammatory response to an experimental stressor compared to healthy controls, suggesting a history of major depression and childhood adversity can sensitize the inflammatory response to psychological stressors. Finally, in a longitudinal study among female adolescents, developing a recent major depressive episode (i.e., over the past 6 months) in those with higher levels of childhood adversity was associated with larger increases in CRP and IL-6 levels compared to when participants were euthymic (Miller and Cole, 2012). Importantly, elevated CRP levels in these individuals persisted 6 months later in the absence of depressive episodes. In this context, Danese et al. (2011) raised an important question of when the effect of exposure to childhood adversity on inflammation emerges. To answer this question, Danese et al. (2011) studied a sample of children at age 12 and found that children with depression who had a history of physical maltreatment exhibited elevated levels of CRP compared to control children (i.e., no depression at age 12 and no history of physical maltreatment). This difference was not evident in depressed-only or maltreated-only children. The findings from this study suggest that the effect of childhood adversity on inflammation among individuals with depression can emerge during the childhood years.

In general, these findings are in line with the concept of neuro-inflammatory sensitization, which links stress, inflammation, and depression (Slavich and Irwin, 2014). The notion of neuro-inflammatory sensitization posits that exposure to early life stress can potentiate the stress response *via* the sympathetic nervous system and the hypothalamus-pituitary-adrenal axis, such that the threshold or magnitude of psychological distress evoking inflammation may be lowered over time (Slavich and Irwin, 2014). Inflammation, in turn, can signal the central nervous system and promote depressive symptoms *via* behavioral and neurobiological alterations. One of these neurobiological alterations include the breakdown of the blood-brain barrier, which filters the connection between the brain and the body. This alteration results from increased levels of intracellular adhesion molecule-1 (ICAM-1), which sits in the endothelium of blood vessels and facilitates the recruitment and entrance of leukocytes into tissues, such as the brain. ICAM-1 can be released from the endothelium as soluble intracellular adhesion molecule-1 (sICAM-1), which then circulates in the blood, which can act as a biomarker for neural-inflammation. Indeed, higher sICAM-1 levels in the blood are associated with the clinical severity of depressive symptoms (Müller, 2019). As a result, the neural-inflammatory pathway may become more robust in depressed individuals with early life stress, such that less stress is required to promote inflammation and inflammation could persist in the absence of actual or perceived stressor (Miller and Cole, 2012; Slavich and Irwin, 2014).

### Susceptibility to Infectious Disease and Response to Vaccines

Several studies have indicated the role of childhood adversity on the immune system’s susceptibility to infection and illness. For instance, Wyman et al. (2007) found that more parental psychiatric symptoms occurring with family stressors were associated with a greater frequency of illnesses in children. Furthermore, greater chronic stress in parents was associated with children’s enhanced natural killer cell function, which functions to recognize cells in the body that have become compromised (i.e., through infection of a virus or conversion to a tumor cell) and kill them to eliminate the threat against the body (Wyman et al., 2007). Caserta et al. (2008) expanded on these findings and similarly found that among children ages 5–10, parental psychiatric symptoms were associated with a higher frequency and rate of illnesses and enhanced natural killer cell function. Clover et al. (1989) found that individuals who perceived their families as dysfunctional had greater rates of influenza B infection than individuals who perceived their families as balanced (as measured by adaptability and cohesion). In another study, Boyce et al. (1995) found that stress was associated with higher respiratory illness rates among children who reported high levels of reactivity to a laboratory or naturalistic stressor, while children with lower levels of reactivity did not exhibit a relationship between stress and illness rates. Wright et al. (2002) examined a potential mechanism between parental stress and respiratory illness and found that higher parental stress prospectively predicted wheezing among infants. Specifically, they hypothesized that parental stress could cause wheezing in infants by impacting the immune system. However, they found that parental stress predicted infant wheezing even after controlling for potential mediators such as maternal smoking, allergen exposure, and increased susceptibility to lower respiratory infections.

Low socioeconomic status during childhood and adolescence has also been associated with greater susceptibility to infectious diseases, such as the common cold in adulthood (Cohen et al., 2004, 2013; Miller et al., 2009; Ziol-Guest et al., 2012). Cohen et al. (2004, 2013) found that childhood socioeconomic status, measured by the number of years during childhood that an individual’s parents owned their homes, was inversely related to an individual’s cold susceptibility in adulthood. However, recent work has shown that high relationship quality with one’s parents, measured by parental care, love and support, lack of conflict with parents, and family cohesiveness, buffered the relationship between low childhood socioeconomic status and susceptibility to the common cold (Cohen et al., 2020).

Stress and other psychosocial factors can blunt the body’s response to vaccines (Madison et al., 2021), yet few studies have explicitly looked at the role of early life stress on vaccine responses. One review by Hayward et al. (2020) postulated that early life stress could modify vaccine response through its effect on mental health. Additionally, O’Connor et al. (2015) showed that negative and hostile interactions observed in a laboratory task between a parent and child predicted a weaker response to the meningococcal vaccine up to 6 months later. During the COVID-19 pandemic, the interaction between early life stress and vaccination immune response will be an important future direction to pursue.

### Latent Herpesvirus Reactivation

Research has also demonstrated an important relationship between childhood adversity and partial latent virus reactivation, and more broadly, the role of early life stress and cellular immune function. Herpes simplex virus (HSV), Epstein–Barr virus (EBV), and cytomegalovirus (CMV) are prevalent herpesviruses that can enter a latent state after infection and then reactivate when the immune system is dysregulated. In other words, after individuals are infected with one of these viruses, the virus remains latent in their body, but if their cellular immune response is compromised, for instance, due to stress, the virus can be reactivated, as reflected by increased antibody titers to the virus (Glaser et al., 1991; Stowe et al., 2010). Thus, early life stress can promote vulnerability to herpesvirus reactivation, reflecting poorer cellular immune system control over the latent virus (Stowe et al., 2010).

Several studies have implicated the relationship between early childhood stress and herpes virus reactivation. Shirtcliff et al. (2009) examined two different types of adverse childhood experiences, institutionalization, and physical abuse, and found higher antibody titers to herpes simplex type-1 (HSV-1) among both adolescents who had been institutionalized and adolescents who had been abused compared to control participants, highlighting a worse cellular immune response over the latent virus among individuals who had experienced an adverse childhood experience. Furthermore, McDade et al. (2000) found that adolescent girls who had experienced trauma had higher EBV antibody titers than girls who had not experienced trauma. Another study showed that individuals who experienced separation from their parents followed by adoption had higher CMV titers than control participants who were not adopted (Elwenspoek et al., 2017). Similarly, among prior EBV-infected adolescents, those who moved into a new parent/caregiver household in early childhood displayed an estimated 100% increase in EBV DNA shedding (Schmeer et al., 2019). In another study, parental psychiatric symptoms were associated with increased CMV-specific T cell activity among CMV seropositive children (Caserta et al., 2008).

Researchers have also examined the role of childhood socioeconomic status and herpesvirus latent reactivation. For instance, living in poverty was associated with elevated CMV antibody titers among children and adolescents ages 11–16 (Dowd et al., 2012). Furthermore, Slopen et al. (2013) examined both the role of childhood socioeconomic status and abuse and found that among adolescents, lower parental occupational status and some categories of lower education were associated with higher levels of EBV antibody titers and that adolescents who had experienced sexual abuse more than ten times had more EBV antibody titers compared to adolescents who had not reported sexual abuse. Furthermore, among individuals who had experienced physical abuse, those who initially experienced physical abuse at ages 3–5 had higher EBV antibodies than those who experienced physical abuse during adolescence (Slopen et al., 2013).

Other studies have examined the relationship between childhood adversity and latent herpesvirus reactivation among adults, demonstrating the role of early experiences on cellular immune responses to the latent virus across the lifespan. For instance, Janicki-Deverts et al. (2014) found that poorer childhood environments, reflected by childhood socioeconomic status, the physical environment, and family relationships, were associated with greater odds of infection with CMV and higher CMV antibody levels among CMV positive participants. Specifically, fewer years of parental homeownership, having a parent who smoked and living in a poorly maintained or unsafe neighborhood were independently associated with greater odds of infection with CMV. Furthermore, among CMV-positive participants, less family warmth, less harmony, more significant dysfunction, and poorer parental bonding were independently associated with higher CMV antibody levels (Janicki-Deverts et al., 2014). In another study, Fagundes et al., 2013b found that experiences of childhood adversities were associated with higher EBV and CMV titers among breast cancer survivors.

### Tumor Environment

In addition to increasing susceptibility to illness, infection, and viruses, early life stress can also affect the immune response to some tumor types. Certain cancers, such as basal cell carcinoma (the most common form of skin cancer), are classified as immunogenic, as the immune system plays an important role in the progression of the tumors (Fagundes et al., 2012). Research on basal cell carcinoma patients who had experienced a severe life stressor in the past year showed that patients who had experienced emotional childhood maltreatment by their mothers or fathers were more likely to have a poorer immune response to the basal cell carcinoma tumor, as reflected by lower levels of messenger RNA (mRNA) coding for immune markers associated with basal cell carcinoma tumor progression and regression, i.e., CD25 (a component of the interleukin (IL)-2 receptor on lymphocytes), CD3ε (a component of the T-cell receptor), ICAM-1 (a cell membrane glycoprotein expressed in both endothelial cells and leukocytes), and CD68 (a cell membrane glycoprotein found in monocytes and macrophages) (Fagundes et al., 2012). However, these findings did not hold among individuals who had not experienced a severe life stressor in the last year, implicating that early adversity may particularly impact those who have recently experienced severe stress, potentially due to a hypersensitivity to stress among individuals who have faced early adversity.

There is work highlighting the relationship between childhood adversity and inflammation among breast cancer survivors. Although the clinical relevance of findings such as this is ambiguous, elevated inflammation is associated with breast cancer recurrence and post-treatment cancer-related fatigue (Bower, 2007; Tabassum and Polyak, 2015; Fagundes et al., 2017). Childhood adversity, measured by abuse, neglect, and a chaotic home environment, was associated with inflammatory markers among breast cancer survivors, with a positive association between maltreatment and IL-6, chaotic home environment and IL-6, and a chaotic home environment and soluble TNF receptor type II (sTNF-RII; Crosswell et al., 2014). Furthermore, Janusek et al. (2013) found that experiences of childhood emotional abuse/neglect among women with breast cancer were associated with more significant behavioral symptoms and greater immune dysregulation, as measured by elevated IL-6 over time. Although findings such as these are intriguing, future work is needed to determine if they are clinically relevant.

### Telomere Shortening

There has been mounting interest in examining the role between childhood adversity and telomere length. Telomeres, or the protective cap at the tips of chromosomes play an important role in facilitating cell aging. Chromosomes are structures made up of linear double-stranded DNA, containing genetic information that programs cells with the necessary characteristics for their specific functions. In humans, due to the mechanism of replication of DNA in chromosomes, the cell cannot copy the entirety of the chromosome. With each replication, the cell loses more and more genetic data, which can be fatal for the cell over time. To compensate for the innate loss of genetic data in replication, chromosomes have telomeres on their ends, which are made up of repeating segments of DNA that will not affect the cell if they are lost with each replication cycle. However, eventually, telomeres will shorten over time, which can indicate the cell’s life span (see Calado and Young, 2009 for more background on telomeres). Shorter telomeres are associated with greater risk for earlier mortality (Epel et al., 2004; Mons et al., 2017; Wang et al., 2018) and psychiatric diseases, including depression (Lindqvist et al., 2015). Mechanistically, elevated inflammation can activate T-cell proliferation, leading to the shortening of telomeres (Aviv, 2004; Kiecolt-Glaser and Glaser, 2010). Importantly, a meta-analysis found a significant association between the level of childhood psychosocial stressors and telomere length across 27 samples, including 16,238 participants (Hanssen et al., 2017).

Tyrka et al. (2010) first examined the relationship between early-life stress and shortened telomeres among young adults, such that those who were maltreated as children had shorter telomeres in cells than those who reported no maltreatment. Kananen et al. (2010) further confirmed an association between childhood adversity and shorter telomere length, such that adults who reported more childhood adverse life events had shorter telomeres than those who reported fewer childhood adversities. However, there was no evidence that current psychological distress impacted telomere length. Recent studies and meta-analyses have continued to demonstrate the relationship between childhood adversity and telomere shortening (Tyrka et al., 2016; Ridout et al., 2018; Bürgin et al., 2019). Furthermore, this relationship between childhood adversity and telomere length existed among individuals with anxiety disorders and controls. O’Donovan et al. (2011) found that exposure to childhood trauma was associated with shorter leukocyte telomere length and that experiences of childhood trauma accounted for differences in leukocyte telomere length between individuals with PTSD and controls. Another study examined individuals at baseline (between 6 and 30 months old) and at a 54-month follow up and found that among individuals who had been institutionalized (e.g., in foster care) as young children, those who had been institutionalized for a greater length of time had significantly shorter telomeres than those who were institutionalized for less time (Drury et al., 2012). Drury et al. (2014) found that children with higher exposure to family violence and disruption had significantly shorter telomere length. Longitudinally, Shalev et al. (2013) found that children exposed to more than two forms of violence had shorter telomeres than children who were unexposed or less exposed to violence. Together, Drury et al. (2012, 2014) and Shalev et al. (2013) suggested that the relationship between early adversity and telomere shortening may also depend on the extent to which an individual faces adversity.

Researchers have also demonstrated that the relationship between early adversity and telomere length in older adults (Osler et al., 2016; Puterman et al., 2016). Ämmälä et al. (2021) recently found that adults with three or more childhood adversities had shorter leukocyte telomere length than individuals with 0, 1, or 2 childhood adversities; this effect remained significant after adjusting for known leukocyte telomere length-associated lifestyle and sociodemographic factors. Similarly, Surtees et al. (2011) found that among women ages 41–80, greater exposure to adverse childhood experiences was correlated with shorter telomeres, while current social adversity or emotional health did not have a relationship with telomere length. Furthermore, in a study of older adult family dementia caregivers and non-caregivers, those who experienced childhood adversity had shorter telomeres than those who were not abused, which potentially translates into a 7–15-year difference in lifespan (Kiecolt-Glaser et al., 2011).

Other studies have examined the role of childhood socioeconomic status and telomere length across the lifespan. For instance, among newborns, boys whose parents had a higher socioeconomic status had a longer cord blood telomere length and placental telomere length (Martens et al., 2020). In another study, Needham et al. (2012) measured childhood socioeconomic status by assessing parental education and found that children ages 7–13 with at least one parent who had completed college had longer telomeres than children whose parents did not attend college; this difference was roughly equivalent to 6 years of aging. In a similar study examining a sample of African American participants, Khan et al. (2021) found that participants whose mothers had more education had longer average telomere length. This study also found that 19% of this effect was mediated through the participant’s education level. The relationship between childhood socioeconomic status and telomere length has also been examined among adults. Cohen et al. (2013) found that lower childhood SES, measured by fewer years of parental homeownership, was associated with shorter CD8+CD28- telomere length in adulthood. Thus, research has indicated that childhood socioeconomic status plays a role on telomere length across the lifespan.

## Childhood Adversity and Epigenetic Pathways

Researchers have aimed to understand how epigenetic mechanisms modulate the long-lasting adverse health effects of early adversity for the last decade. The stress associated with early adversity disrupts the regulation of fundamental biological systems during sensitive periods of development, which puts people at risk for a wide range of health problems (Miller et al., 2011). Further, the reprogramming of stress-sensitive gene pathways sensitizes the developing brain to the effects of early stress exposure throughout the lifespan (Bondar and Merkulova, 2016). Environmental programming of life-long phenotypes begins very early in life (Vaiserman, 2015); thus, epigenetic alterations may embed adverse early life experiences in the genome.

Epigenetics is the study of phenomena and mechanisms that cause chromosome-bound, heritable changes to gene expression, independent of DNA sequence changes (Deans and Maggert, 2015). These “phenomena” refer to behavioral and environmental impacts on gene functioning. Human development is controlled by epigenetic mechanisms, which help differentiate and record environmental information and shape cellular and physiological functions across the lifespan, with particularly pronounced effects during fetal development that decrease as age increases. One such mechanism is DNA methylation, or the addition of a methyl group to DNA that typically impairs the expression of a gene (Jones and Takai, 2001; McGowan et al., 2009; Crews, 2010). Higher DNA methylation levels are typically associated with lower rates of gene transcription and, therefore, functioning (Allis and Jenuwein, 2016). These modifications in DNA may underlie the associations between early life stress and physical health problems (McGowan et al., 2009; Crews, 2010).

Substantial evidence derived from research with animal models suggest that environmental programming of life-long phenotypes begins very early in life (e.g., maternal diet; Waterland and Jirtle, 2003). For instance, in animal models, maternal behavior can alter gene expression *via* DNA methylation changes in the offspring. For example, maternal care in rats can affect hippocampal glucocorticoid receptor expression and subsequent HPA function for the offspring (Weaver et al., 2004). Additionally, the maternal effect on glucocorticoid receptor expression and HPA response to stress was mediated by changes in chromatin structure (Weaver et al., 2004). Thus, both changes in methylation and chromatin structure are implicated.

Studies investigating epigenetic markers across the entire human genome, indicating that epigenetic impacts are both long-lasting and transgenerational. For example, Appleton et al. (2013) found that infants whose mothers experienced the greatest levels of socioeconomic adversity during pregnancy had the lowest extent of methylation of the placental 11b-HSD2 gene, indicating lower gene functioning. During prenatal development (pregnancy), environmental cues transmitted from mother to fetus may program an epigenetic response to the infant’s post-natal environment through less cortisol exposure (O’Donnell et al., 2012). Further, experiences of maternal depression and anxiety were associated with poor newborn neurobehavior. Poor neurodevelopmental outcomes are associated with increased methylation of placental genes [i.e., NR3C1 and 11bhydroxysteroid dehydrogenase type 2 (11b-HSD-2)] involved in HPA axis functioning (Conradt et al., 2013). In this way, exposure to environmental stress *in utero* may program an infant’s neurological functioning.

Far beyond fetal development, epigenetics plays an important role in determining the long-term impact of early life adversity. For example, epigenetic and environmental mechanisms are linked to the etiology and pathology of depression and serious negative mental health consequences, such as suicide (for a review, see Lin and Tsai, 2019). For instance, adolescents raised in lower SES environments as children had higher Toll-like receptor 4 (TLR4) mRNA levels than their higher SES counterparts, indicating that early adversity may alter the genes linked to adult immune dysregulation (Miller and Chen, 2007). In a post-mortem sample of people who had died by suicide, McGowan et al. (2009) examined differences in epigenetic regulation of hippocampal glucocorticoid receptor expression by comparing the brains of those with a child abuse history, those with no child abuse history, and those who were victims of sudden, accidental death with no history of abuse (controls). The researchers found decreased hippocampal NR3C1 gene expression in those who died by suicide and had a history of childhood abuse, compared to accidental death victims with no history of abuse. Furthermore, there were no differences in gene expression between suicide victims and accidental death victims *without* a history of childhood abuse, suggesting that changes in glucocorticoid receptor expression may be more closely associated with a history of childhood abuse than with suicide completion. McGowan et al. (2009) cautiously speculate that epigenetic processes might mediate the effects of a stressful early-life environment on hippocampal gene expression. DNA methylation, a stable epigenetic marker, could persist into adulthood and make people vulnerable for psychopathology, potentially *via* HPA activity (McGowan et al., 2009). While more research is still needed, these findings may have important clinical implications. For instance, Bower et al. (2020) utilized genome-wide transcriptional profiling of isolated monocytes and found greater NF-κB-binding motifs within the promoters of up-regulated vs. down-regulated genes in women with breast cancer who experienced childhood maltreatment, compared to women with breast cancer who did not experience childhood maltreatment. These findings held when controlling for depression and indicated greater inflammatory signaling among women with breast cancer who had experienced childhood maltreatment.

Epigenetic regulation may also mediate the relationship between broader environmental influences in early life than just those that tend to occur inside the home (e.g., childhood abuse, parental attentiveness). Recently, Reuben et al. (2020) provided evidence from a large longitudinal cohort study that neighborhood disadvantage is associated with DNA methylation differences in genes involved in inflammation. Continued research on the epigenetic influence of broader early life environments could provide vital information to inform neighborhood-level policy interventions, impacting long-term health among those raised in disadvantaged neighborhoods.

The study of early life adversity and epigenetics is a growing field (Krause et al., 2020). One of the many major challenges of future investigation will be to uncover how specific factors interact with epigenetic mechanisms to promote susceptibility or resilience to the negative effects of early life adversity. For example, individual differences in sex, genetic background, age, and stressor type, timing, and duration may influence the extent to which early life adversity impacts the genome and behavioral outcomes. Furthermore, most studies studied DNA methylation, which is but one specific epigenetic mechanism. Epigenetic mechanisms other than DNA methylation may also contribute to the regulation of stress pathways. Future research should elucidate how different adverse childhood experiences result in epigenetic markers, and whether certain treatments may reverse these markers. For a complete review of epigenetics and early life adversity, see Burns et al. (2018).

## Discussion

In sum, childhood adversity, ranging from child abuse and neglect to poor parent/child relationships, to low socioeconomic status, can negatively impact immune and epigenetic pathways across the lifespan. There are several mechanisms and pathways in which immune and epigenetic dysregulation can emerge, including increased inflammation, susceptibility to illness, latent herpesvirus reactivation, shortened telomeres, DNA methylation, and histone modification. Importantly, these immune and epigenetic dysregulation as a result of childhood adversity can have severe clinical implications including poorer mental health, including increased risk of depression, as well as poorer physical health, including increased risk for cardiovascular, type II diabetes, and cancer in adulthood (Heim et al., 2008; Miller et al., 2011).

Future research should expand upon the work examining early life stress and health outcomes to identify racial disparities that underlie this relationship. It is important to note that early life adversity, including experiences of childhood maltreatment, exposure to crime, and other forms of external stressors, disproportionately affect racial/ethnic minorities (Suglia et al., 2020). For instance, African American and Hispanic youth have exhibited an increased risk of low-grade inflammation compared to White youth (Schmeer and Tarrence, 2018). Additionally, Ford and Stowe (2013) found that Black/African American youth had significantly higher EBV antibody levels than White youth. These disparities are increasingly understood as related to the chronic stress experienced by racial/ethnic minorities (Suglia et al., 2020). Future work should continue to examine how racial disparities can underlie the relationship between early adversity and poor health outcomes throughout the lifespan.

The contribution of early-life food insecurity to health across the lifespan is an important area for future research. Food insecurity generally may promote inflammation in several ways: (1) by promoting central adiposity (Salinas et al., 2016), (2) through psychosocial stress (Bermúdez-Millán et al., 2016), and (3) from a diet that directly promotes inflammation (Bergmans et al., 2018). Salinas et al. (2016) found that food insecurity, especially when food insecurity was assessed through a question focused on not having money to buy balanced meals, was associated with waist circumference for 554 Mexican-American women, which indicates that food insecurity promotes central adiposity. In a sample of 121 low-income Latino adults with type 2 diabetes mellitus, Bermúdez-Millán et al. (2016) identified that food insecurity was associated with suboptimal sleep quality and that this effect was mediated by several measures of psychological distress (i.e., depressive symptoms, anxiety symptoms, and diabetes distress); thus, food insecurity can be a significant source of distress and this distress is associated with worse sleep. In a sample of nearly 11,000 lower-income adults in the United States, Bergmans et al. (2018) identified that higher levels of food insecurity were associated with a higher Dietary Inflammatory Index (i.e., a diet that potential induces inflammatory pathways).

Despite the strong evidence of the relation between food insecurity and adult health, there is less research that examines the consequences of food insecurity in childhood on health across the lifespan. In addition to the previously addressed pathways from food insecurity to inflammation, early-life food insecurity may also affect neurodevelopment in a manner that has proven difficult to disentangle from poverty generally (Rosales et al., 2009; Johnson et al., 2016). However, in a study of approximately 11,500 children under 36 months of age, those who were food insecure were nearly 3× as likely to have been hospitalized since birth (Cook et al., 2004). Importantly, food stamps attenuated the relationship between increased food insecurity and caregiver reports of worse child health (Cook et al., 2004), which emphasizes both the importance and feasibility of using policy to reduce the impact of food insecurity on child health.

Given the ample research on immune and epigenetic pathways linking childhood adversity to health outcomes across the lifespan, future directions in this field should seek to develop, test, and implement interventions for those who have experienced early life stress. To develop interventions targeting the impact of early life stress and immune and epigenetic dysregulation, understanding the implications of attachment theory on these pathways can help develop targeted interventions with a theoretical basis. Attachment theory suggests that an individual’s troubled relationships with their parents can lead to a developed sense of emotional insecurity that has lasting consequences on how an individual copes with relational stress and navigates close relationships across the lifespan (Thompson, 2008; Mikulincer and Shaver, 2009). An individual’s attachment orientation can serve as an indirect pathway to adverse health outcomes following early-life adversity (Maunder et al., 2017; Widom et al., 2018). For example, in a sample of hospital workers and paramedics, researchers found a significant indirect effect of early life adversity on physical symptoms through attachment anxiety, a type of attachment insecurity (Maunder et al., 2017). Thus, individuals who experience childhood adversity, such as child abuse or neglect, may develop insecure attachment, contributing to poor health outcomes across the lifespan. Furthermore, there has been work examining the relationship between attachment insecurity and immune dysregulation, including increased inflammation (Ehrlich et al., 2019; Gouin and MacNeil, 2019), shortened telomere length (Murdock et al., 2018a,b), and latent herpesvirus reactivation (Fagundes et al., 2014). For example, Fagundes et al. (2014) found that individuals with higher attachment anxiety also had higher EBV VCA IgG antibody titers than those with less attachment anxiety.

Indeed, individuals who are insecurely attached are more physiologically sensitive to stress (reflected by greater cortisol increases in response to stress) than those with secure attachment (Diamond, 2001; Laurent and Powers, 2007; Diamond et al., 2008; Rifkin-Graboi, 2008; Diamond and Fagundes, 2010; Fagundes et al., 2011). Increased cortisol in response to stress is in accord with research linking poor early parental experience and more pronounced stress-induced glucocorticoid production (Heim et al., 2000, 2008; Sanchez, 2006). Importantly, increased cortisol sustained over time can lead to glucocorticoid insensitivity, which can, in turn, increase inflammation *via* the production of proinflammatory cytokines (Miller et al., 2002). Accordingly, the relationship between childhood adversity (e.g., child abuse and neglect, poor parent-child relationships, and family conflict) and inflammation may also be related to the association between attachment style and inflammation.

Based on findings that arose using animal models of maternal care in rats, there has been an experimental shift in human attachment research to focus on gene-by-environment interaction by epigenetic mechanisms (Champagne, 2008; Ein-Dor et al., 2018). Over many decades of attachment research, researchers documented a “transmission gap” of “maternal responsiveness” that can partly explain the intergenerational transmission of attachment (Van Ijzendoorn and Bakermans-Kranenburg, 1997). For example, epigenetic modification of the oxytocin and glucocorticoid receptor genes is linked to attachment avoidance in young adults (Ein-Dor et al., 2018). Indeed, studies show a direct link between attachment and DNA methylation (Van Ijzendoorn et al., 2010; Mulder et al., 2017; Bosmans et al., 2018). For instance, more stressed children with less maternal support report more significant attachment anxiety when their NR3C1 gene is highly methylated (Bosmans et al., 2018). Given the role that attachment insecurity can have on immune and epigenetic pathways (Maunder et al., 2017; Widom et al., 2018), understanding the implications of attachment theory on immune and epigenetic pathways can be useful in understanding the broader relationship of childhood adversity and health across the lifespan.

While there have been intervention programs targeting the impact of childhood adversity broadly, including programs for foster care parents and general therapies for parents and children (Shonkoff, 2016; Turner et al., 2016; Boparai et al., 2018), it is worth noting the utility of interventions that can address the impacts of childhood adversity across the lifespan. Furthermore, when developing interventions, it is efficacious to utilize a known theoretical framework to best target and operationalize specific points of intervention. Targeted interventions using attachment theory are especially effective in both adolescents facing adversity (e.g., incarceration; Keiley, 2002) and can continue to be useful even among adults (Bifulco and Thomas, 2012). Importantly, attachment theory has also been utilized among individuals who have experienced trauma (Muller, 2009), further providing evidence on the utility of using attachment theory as a framework that can be used to develop targeted interventions to those who have experienced childhood adversity, including child abuse and neglect, family conflict, and poor parent/child relationships. The field has come a long way in demonstrating the relationship between childhood adversity and health and immune and epigenetic pathways across the lifespan that contribute to this relationship. Thus, future directions in the field of childhood adversity and health should focus on developing targeted interventions for individuals across the lifespan that can mitigate adverse health outcomes in young children and older populations who continue to be impacted by the long shadow of childhood adversity.

## Author Contributions

MC wrote the introduction and discussion section and reviewed the majority of the childhood adversity and immune section. AL wrote the epigenetic pathways section. MM wrote the Early Life Stress, Inflammation, and Depression section. JC contributed to the writing of the inflammation section and telomere section. RB contributed to the conceptualization of early-life stress related to attachment theory/parental experiences and racial disparities. LC contributed to the vaccine portion of the manuscript and the overall conceptualization of the manuscript. CF contributed to the writing of all sections, giving thorough feedback at each level and playing a heavy role in the conceptualization of the manuscript and writing portions of each section. All authors have read and approved the final manuscript and contributed to the writing of this review.

## Conflict of Interest

The authors declare that the research was conducted in the absence of any commercial or financial relationships that could be construed as a potential conflict of interest.

## Publisher’s Note

All claims expressed in this article are solely those of the authors and do not necessarily represent those of their affiliated organizations, or those of the publisher, the editors and the reviewers. Any product that may be evaluated in this article, or claim that may be made by its manufacturer, is not guaranteed or endorsed by the publisher.
